# Paraquat (Pesticides)

**DOI:** 10.14252/foodsafetyfscj.D-22-00012

**Published:** 2022-12-23

**Authors:** Food Safety Commission of Japan

## Abstract

Food Safety Commission of Japan (FSCJ) conducted a risk assessment of a bipyridinium herbicide, paraquat (CAS No. 1910-42-5), based on results from various studies. Major adverse effects of paraquat in experimental animals were observed in body weight (suppressed weight gain), lungs (increased weight, alveolar epithelium hyperplasia, and pneumonia), kidneys (renal tubule dilatation) and eyes (cataract in rats and dogs). The effects on the lung were considered to be the most critical endpoints in the assessment. Neither carcinogenicity, effects on fertility, teratogenicity, genotoxicity, or immunotoxicity was observed. FSCJ reasonably concluded no obvious concern of paraquat-residue in foods to yield neurotoxicity through human dietary exposure, as long as paraquat is applied following the registered standard use of the pesticide. The lowest no-observed-adverse-effect level (NOAEL) obtained from all the studies was 0.45 mg paraquat ion^*^/kg bw per day in one-year chronic toxicity study in dogs. FSCJ specified an acceptable daily intake (ADI) of 0.0045 mg paraquat ion/kg bw per day by applying a safety factor of 100 to the NOAEL. FSCJ judged these effects also as the end-point of the acute reference dose (ARfD). The lowest NOAEL was 0.45 mg paraquat ion/kg bw per day in one-year chronic toxicity study in dogs. For potential adverse effects of a single oral administration of paraquat, FSCJ specified an ARfD to be 0.0045 mg paraquat ion/kg bw by applying a safety factor of 100 to the NOAEL.

## Conclusion in Brief

Food Safety Commission of Japan (FSCJ) conducted a risk assessment of a bipyridinium herbicide, paraquat (CAS No. 1910-42-5), based on results from various studies.

The data used in the assessment includes the fate in animals (rats, mice, cattle, chicken and others), fate in plants (lettuce and soy beans and others), residues in crops, and tests of acute neurotoxicity (rats), subacute toxicity (rats, mice and dogs), subacute neurotoxicity (rats), chronic toxicity (rats and dogs), combined chronic toxicity/carcinogenicity (rats and mice), carcinogenicity (mice), two- and three- generation reproductive toxicity (rats), developmental toxicity (rats and mice), genotoxicity and immunotoxicity (rats). Scientific findings in human are also included.

The major adverse effects of paraquat in experimental animals were observed in body weight (suppressed weight gain), lungs (increased weight, alveolar epithelium hyperplasia, and pneumonia), kidneys (renal tubule dilatation) and eyes (cataract in rats and dogs). The effects on the lung and respiratory organs were considered to be the most critical endpoints in the assessment. Neither carcinogenicity, effects on fertility, teratogenicity, genotoxicity, or immunotoxicity was observed. After reviewing of currently available results of non-clinical studies and scientific findings in human, FSCJ reasonably concluded no obvious concern of paraquat-residue in foods to yield neurotoxicity through human dietary exposure, as long as paraquat is applied following the registered standard use of the pesticide.

Based on the results from various studies, paraquat (parent compound only) was identified as the substance relevant to the residue definition for dietary risk assessment in agricultural products and livestock products.

The lowest no-observed-adverse-effect level (NOAEL) obtained from all the studies was 0.45 mg paraquat ion^*1^/kg bw per day in one-year chronic toxicity study in dogs. FSCJ specified an acceptable daily intake (ADI) of 0.0045 mg paraquat ion/kg bw per day by applying a safety factor of 100 to the NOAEL.

The sensitive and critical endpoints of paraquat after the oral administration appeared on the lung and respiratory organs throughout the studies examined. Effects on the lung were detected even in animals of impending sacrifice and of dead state in acute toxicity studies. These data suggested the gradual time dependent deterioration of the lung after the paraquat exposure. Thus, paraquat was possible to initiate the lung and respiratory organ damages after single exposure, which is consistent with the histopathological findings in the repeated dose study. Accordingly, FSCJ judged the consequence as the end-point of the acute reference dose (ARfD). The lowest NOAEL was 0.45 mg paraquat ion/kg bw per day in one-year chronic toxicity study in dogs. For potential adverse effects of a single oral administration of paraquat, FSCJ specified an ARfD to be 0.0045 mg paraquat ion/kg bw by applying a safety factor of 100 to the NOAEL.

## Acknowledgement

## Appendix

### FSCJ’s View on Paraquat Neurotoxicity*^2^

No neurotoxicity was detected in acute and 90-day subacute studies of paraquat in rats, so far surveyed^1^^)^. Neurotoxicity attributed to paraquat was not observed in other toxicological studies.

Paraquat is structurally similar to a dopaminergic neurotoxic substance, 1-methyl-4-phenyl-1, 2, 3, 6-tetrathydropyridine (MPTP). To investigate the possible causal relationship between paraquat and Parkinson disease, various nonclinical experiments had been done^1^^)^.

JMPR, EPA and APVMA concluded, from the available information on the toxicities of paraquat, no concern of neurotoxicity through human dietary exposure to paraquat as pesticide residues in food, and also effects on lung as the most critical endpoints even after dietary intakes.

The rationales are summarized below.

(1) MPTP is transported readily across the blood-brain barrier to get in the brain. MPTP is then converted to MPDP^+^ (1-methyl-4-phenyl-4-phenyl-2,3-diydropyridinium) and oxidized into the toxic cation MPP^+^ (1-methyl-4-phenylpyridinium) to cause dopaminergic neurotoxicity. Although an involvement of dopamine transporter is suggested for paraquat^2^^)^, this di-cation chemical is not readily taken up into the brain^3^^)^.

(2) Behavioral, neurological and/or neuropathological effects were detected after subcutaneous, intraperitoneal and intracerebral administrations of paraquat. Inconsistencies were, however, observed within the experimental data.

Due to the difference in the route of administration and tissue distribution, these data have only limited relevances to human exposure through food intakes of paraquat as pesticide residue.

(3) In properly designed tests including positive control groups, no behavioral, neurological or neuropathological effects were observed after the oral administration^1^^,^^3^^)^.

Some epidemiological studies suggested the association between paraquat exposure and Parkinson disease. The rationale for such relationship is, however, not yet established from the points of methodologies related to research design, statistical power, diagnostic criteria, exposure assessment, bias, and confounding factors. Enhanced risk of Parkinson disease was not observed in paraquat manufacturing workers. No case report was also available for Parkinson disease symptoms (bradycardia, tremor, stiffness and posture instability) in short or long-term survivors who were exposed to paraquat and manifesting its effects on the lungs.

FSCJ concluded no concern of neurotoxicity of paraquat in food based on the currently available non-clinical test results and scientific findings in human, as long as the pesticide is used following the registered standard of use. This conclusion is consist with the views of overseas institutions.

**Table 1.**
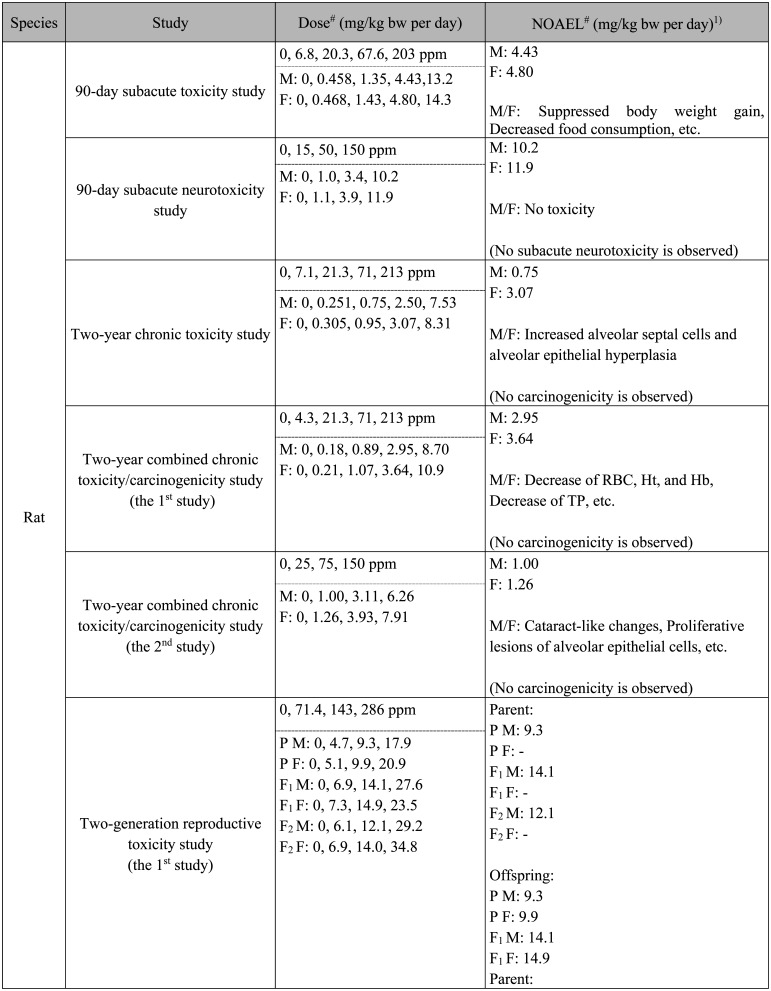
Levels relevant to toxicological evaluation of paraquat

**Table 1.**
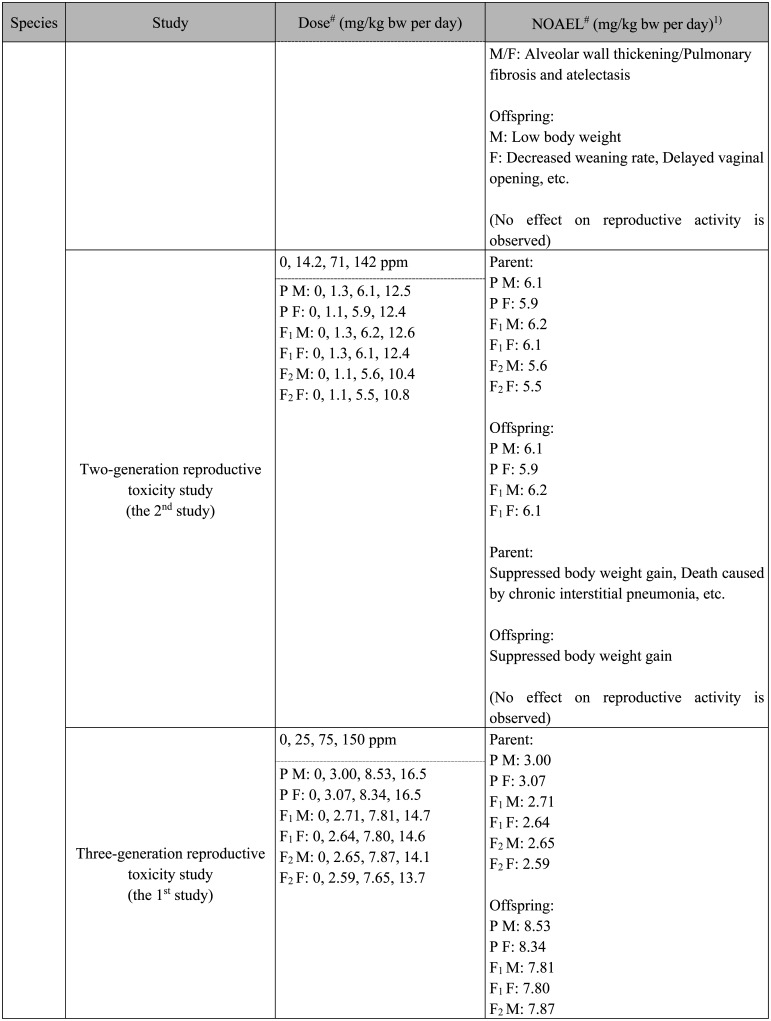
Continued

**Table 1.**
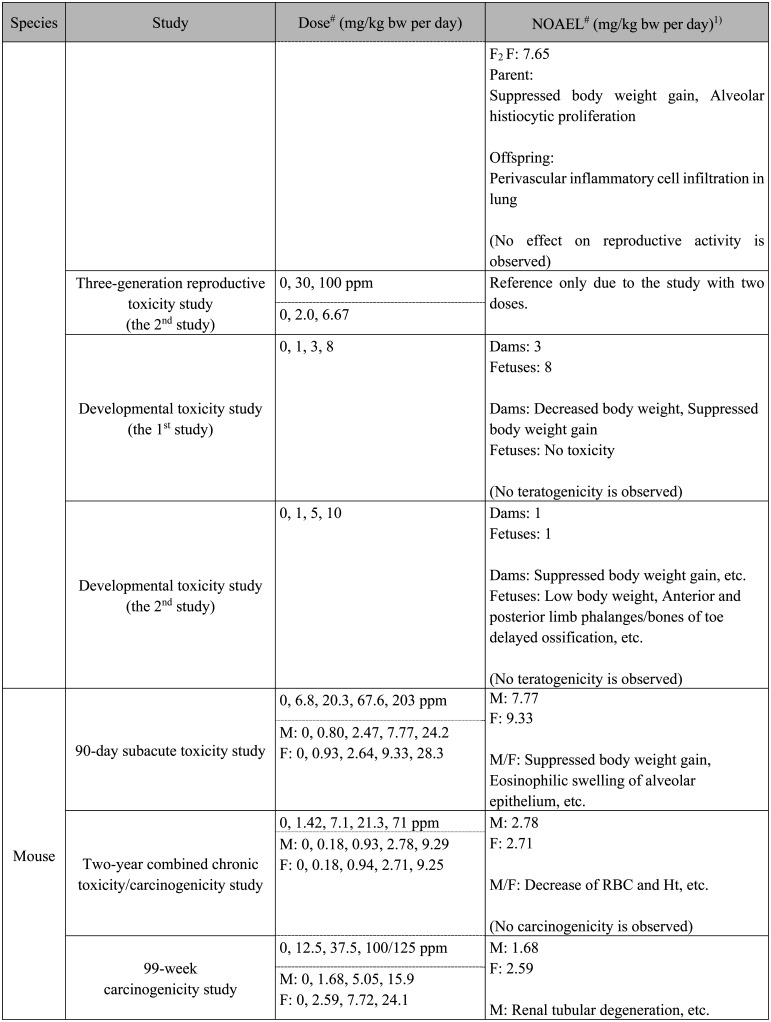
Continued

**Table 1.**
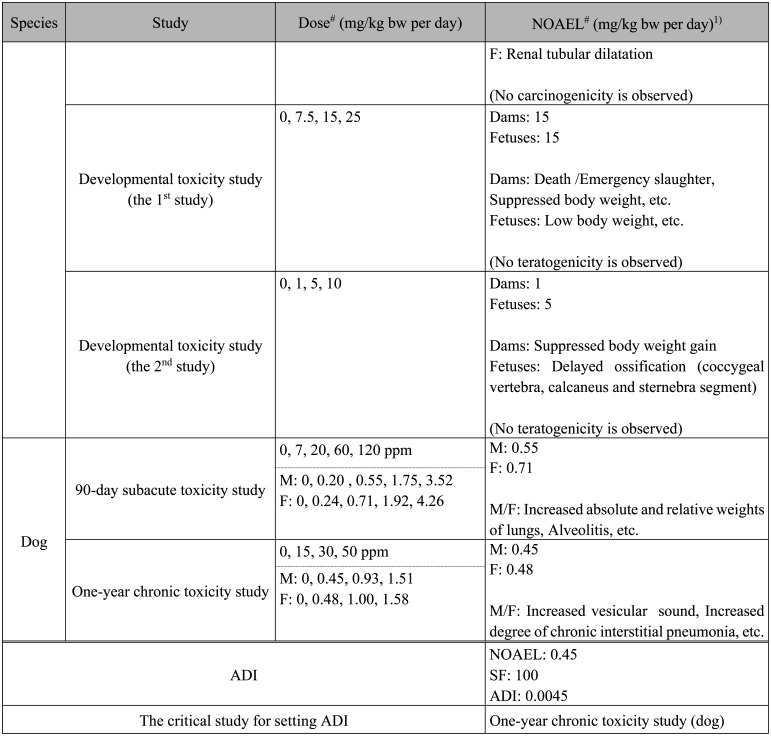
Continued

ADI: Acceptable daily intake, SF: Safety factor, NOAEL: No-observed-adverse-effect level

-: NOAEL could not be specified.

^#^: Converted value for paraquat ion

^1)^ The adverse effect observed at LOAEL

**Table 2.**
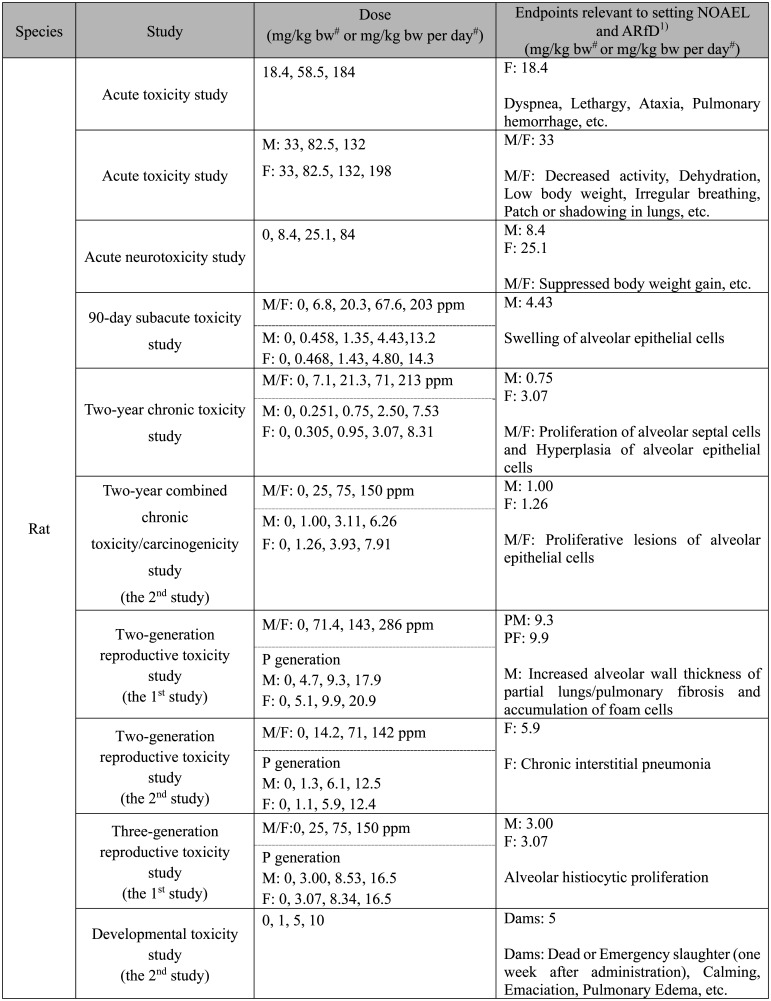
Potential adverse effects of a single oral administration of paraquat

**Table 2.**
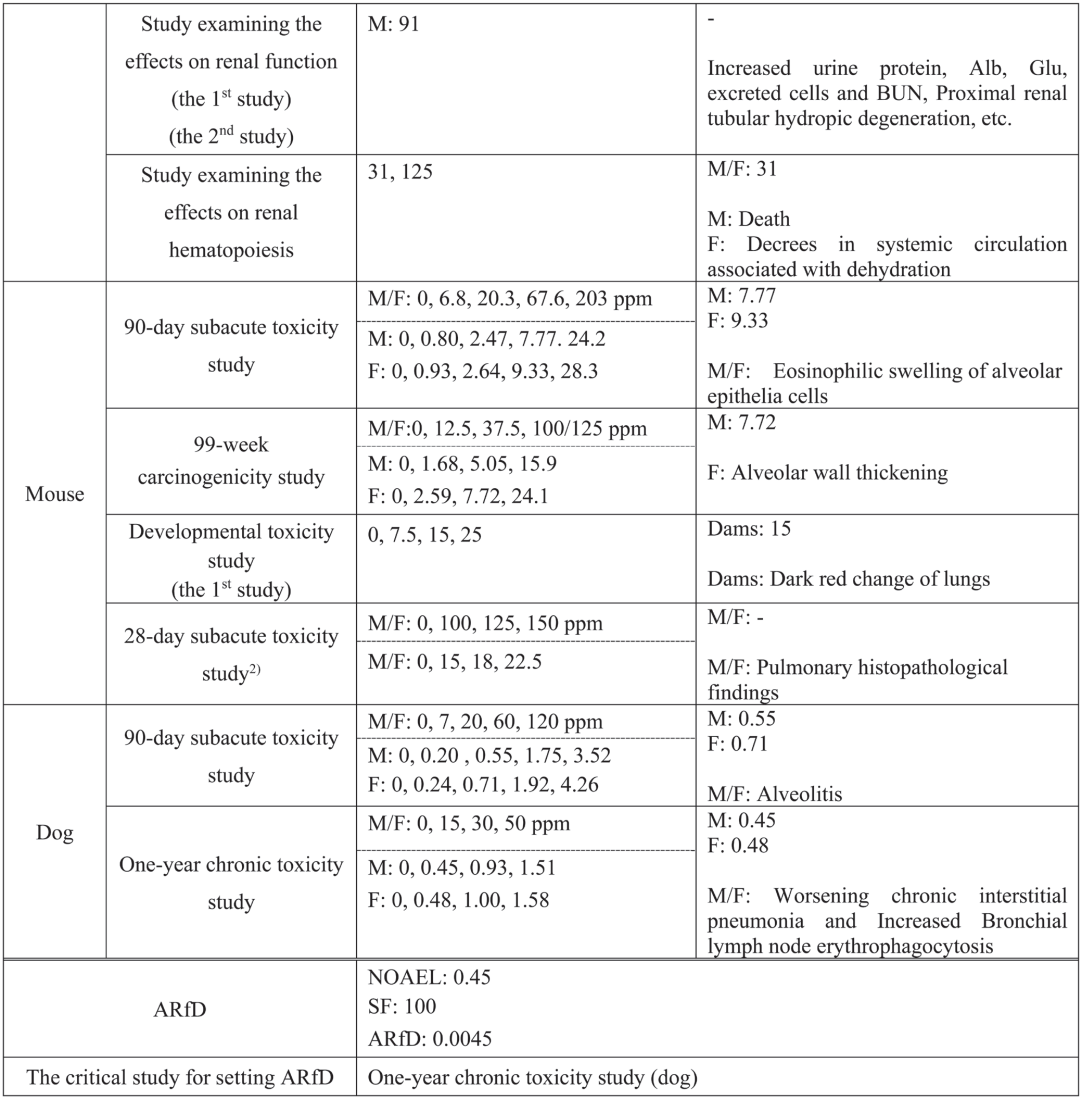
Continued

ARfD: Acute reference dose, NOAEL: No-observed-adverse-effect level, SF: Safety factor

-: NOAEL could not be specified.

^#^as a value converted for paraquat ion.

^1)^The adverse effect observed at LOAEL

^2)^Study for differences of toxic effects using manufacturing grade and technical grade active ingredients of paraquat.
